# Phenotypic Microdiversity and Phylogenetic Signal Analysis of Traits Related to Social Interaction in *Bacillus* spp. from Sediment Communities

**DOI:** 10.3389/fmicb.2017.00029

**Published:** 2017-01-30

**Authors:** María Dolores Rodríguez-Torres, África Islas-Robles, Zulema Gómez-Lunar, Luis Delaye, Ismael Hernández-González, Valeria Souza, Michael Travisano, Gabriela Olmedo-Álvarez

**Affiliations:** ^1^Laboratorio de Biología Molecular y Ecología Microbiana, Departamento de Ingeniería Genética, Unidad Irapuato, Centro de Investigación y de Estudios Avanzados del Instituto Politécnico NacionalIrapuato, México; ^2^Laboratorio de Evolución Molecular y Experimental, Departamento de Ecología Evolutiva, Instituto de Ecología Universidad Nacional Autónoma de MéxicoMexico City, México; ^3^Department of Ecology, Evolution and Behavior, University of Minnesota, Saint PaulMN, USA

**Keywords:** microbial communities, phylogenetic signal, phenotypic trait loss, collective action, *Bacillus* spp.

## Abstract

Understanding the relationship between phylogeny and predicted traits is important to uncover the dimension of the predictive power of a microbial composition approach. Numerous works have addressed the taxonomic composition of bacteria in communities, but little is known about trait heterogeneity in closely related bacteria that co-occur in communities. We evaluated a sample of 467 isolates from the Churince water system of the Cuatro Cienegas Basin (CCB), enriched for *Bacillus* spp. The 16S rRNA gene revealed a random distribution of taxonomic groups within this genus among 11 sampling sites. A subsample of 141 *Bacillus* spp. isolates from sediment, with seven well-represented species was chosen to evaluate the heterogeneity and the phylogenetic signal of phenotypic traits that are known to diverge within small clades, such as substrate utilization, and traits that are conserved deep in the lineage, such as prototrophy, swarming and biofilm formation. We were especially interested in evaluating social traits, such as swarming and biofilm formation, for which cooperation is needed to accomplish a multicellular behavior and for which there is little information from natural communities. The phylogenetic distribution of traits, evaluated by the Purvis and Fritz’s D statistics approached a Brownian model of evolution. Analysis of the phylogenetic relatedness of the clusters of members sharing the trait using consenTRAIT algorithm, revealed more clustering and deeper phylogenetic signal for prototrophy, biofilm and swimming compared to the data obtained for substrate utilization. The explanation to the observed Brownian evolution of social traits could be either loss due to complete dispensability or to compensated trait loss due to the availability of public goods. Since many of the evaluated traits can be considered to be collective action traits, such as swarming, motility and biofilm formation, the observed microdiversity within taxonomic groups might be explained by distributed functions in structured communities.

## Introduction

Communities are assemblages of different species in which organisms co-exist and interact within a given environment. Molecular strategies have been instrumental in uncovering the great taxonomic diversity of microbial communities. Today, however, one of the fundamental pursuits in microbial ecology is to understand what the taxonomic classification of an organism means at the functional level. Thus, simply knowing “who is there” is no longer the most relevant question. The relationship between phylogeny and predicted functional traits revealed great heterogeneity that limits the predictive power of a microbial composition approach. Most previous studies showed trait consistency to the phylum level (Philippot et al., 2010; Goldfarb et al., 2011; Koeppel and Wu, 2012). However, some ecological traits may be species- or strain-specific since microbial genomes are highly dynamic and can change rapidly through loss or acquisition of genes from distant lineages via horizontal gene transfer (HGT, Boon et al., 2014). Also, the gene content of strains within a given species may differ by up to 30–35% (Konstantinidis and Tiedje, 2005). Bacteria species are therefore considered to be a mosaic of transferred genes since their great genetic diversity is obtained from distantly related organisms (Ochman et al., 2000). Differences in lifestyle correlate with variations in genes that are required for interactions with specific environments (Ochman et al., 2000; Polz et al., 2013). Capabilities for nutrient uptake, such as the capability to grow on different carbon sources, have been shown to be easily transferred or to have evolved rapidly from one function to another, probably because few genes are involved (Martiny et al., 2013). Therefore, these traits have been observed to be taxonomically dispersed, and move at shallow depths in phylogenies (Martiny et al., 2013). In contrast, traits associated with complex functions, such as photosynthesis and methanogenesis, are found only in a few deep clades (Martiny et al., 2013).

Community ecology investigates the complex interactions between organisms and the ecological and evolutionary consequences of sharing in a community. In the context of community, genes and functions can be lost when they are no longer needed in the habitat or when their function can be performed by community members such that the production of public goods is sufficient to support community stability (Visser et al., 2010; McInerney et al., 2011; Morris et al., 2012; Boon et al., 2014). Another fundamental aspect of microbial communities is social interactions among cells. At an individual level, many phenotypes affect their neighbor’s environment and thus influence their growth and reproduction. Important ecological processes, in fact, rely on a range of social traits such as biofilms, swarming, and quorum sensing, which are conserved in deep clades (Daniels et al., 2004). Such traits are considered to be collective action traits as they act at group-level (Stoodley et al., 2002; Daniels et al., 2004). Biofilm formation is dependent on the expression of costly exopolymers while swarming requires the production of biosurfactants and extracellular DNA (Velicer and Yu, 2003; Daniels et al., 2004; Spoering and Gilmore, 2006). These secreted substances can be considered as public goods. Swimming, swarming, and biofilm formation are traits that are also deeply rooted among the Firmicutes (Stoodley et al., 2002; Daniels et al., 2004). These traits are typically observed and have been characterized, in the extensively studied soil and pathogenic *B. subtilis* and the *B. cereus* groups. *B. subtilis* displays multicellular and social features, such as swarming and biofilm formation (González-Pastor et al., 2003). These traits can be assumed to be a fixed trait of *Bacillus* spp. and are rarely evaluated in wild isolates.

Swimming motility depends on flagella of individual cells (unorganized movement) that move independently upon perceiving chemical signals (Calvio et al., 2005). On the other hand, swarming is a multicellular behavior that allows rapid movement of differentiated bacterial cells (Kearns, 2010), and depends not only on the synthesis of flagella but also on the secretion of surfactants (Cosmina et al., 1993; Kearns and Losick, 2003). Both swarming ability and biofilm formation provide benefits at a group level since they depend on the coordinated expression of genes and signals (Stoodley et al., 2002; Daniels et al., 2004).

Many studies have focused on the evolution of social traits (Crespi, 2001; Foster et al., 2006; West et al., 2006; Diggle et al., 2007; de Vargas et al., 2011). A study by Whitham et al. (2006) provides a framework to understand how genetically based traits can have an influence at the species level and up to the ecosystem level; this hypothesis is supported by studies of multi-level selection and the fitness consequences of indirect genetic effects. Most efforts to address trait evolution were made in laboratory systems and thus there is very little information regarding trait heterogeneity in bacteria from natural communities, and, in particular, traits that are involved in social interaction.

The Cuatro Cienegas Basin (CCB), geographically isolated by high mountains, is located in the Chihuahuan desert. The CCB harbors a large number of springs among its water systems, and the area features high biological richness, numerous endemisms, and an enormous microbial diversity (Souza et al., 2012). We have systematically studied the diversity of bacteria in this region, particularly in the Churince water system. A genus we have extensively studied is the *Bacillus*, easily isolated due to their thermo-resistance property. The *Bacillus* spp. are abundant in this system and possess traits that correlate with an oligotrophic, phosphorus-limited environment (Tapia-Torres et al., 2016). HGT appears to be involved in the acquisition by *Bacillus coahuilensis* of genes from cyanobacteria that are involved in phosphorous utilization and adaptation to high-light environments (Alcaraz et al., 2008). We previously showed that *Bacillus* spp. from sediment communities are not assembled randomly and that biotic interaction among *Bacillus* members (specifically antagonism) have played a role in shaping community structure in this system (Pérez-Gutiérrez et al., 2013). The *Bacillus* spp. collection from the different sampling sites in this water system has therefore become an excellent model to understand bacterial interactions in natural communities.

In this work, we evaluated phenotypic heterogeneity and phylogenetic signal of phenotypic traits that are known to diverge within small clades and traits that are conserved deep in the lineage. Our results suggest that despite co-occurring in the same communities, different *Bacillus* species have different ecological strategies. The explanation to the observed evolution of these traits could be either loss due to complete dispensability of the trait, or compensated trait loss due to the availability of public goods. The observed microdiversity within taxonomic groups could be explained by distributed functions in structured communities.

## Materials and Methods

### Study Site

The Churince water system is located on the west side of CCB, and is formed by a spring, an intermediate lagoon, and a desiccation lagoon. The water and sediment samples described in this work were obtained from the intermediate lagoon. Physical and chemical measurements revealed homogeneous conditions along the sampling points, including pH (7.8–8), salinity (∼1.7–2.1 ppt), and daily temperature fluctuation (from 20 to 35°C; Pérez-Gutiérrez et al., 2013).

### Bacterial Sampling and Isolation

**FIGURE 1:**
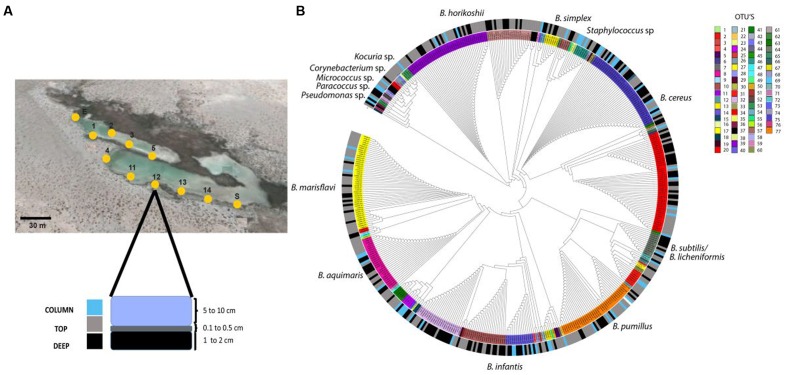
**Spatial distribution of *Bacillus* isolated from the Churince System.** **(A)** Map of sampling location in the Churince water system and method for sampling water (COLUMN), superficial sediment (TOP) and subsuperficial sediment (DEEP); **(B)** Phylogenetic reconstruction of thermo-resistant isolates sequences and their association habitat. The inner ring shows the OTU’s from the different sites (27 for COLUMN, 53 for TOP and 32 for DEEP). The outer ring shows the habitat (blue, water column; gray, TOP sediment; black, DEEP sediment). Map obtained from Google Maps (2013).

The isolates in the collection were obtained in October 2007 from water column and sediment samples of the Intermediate Lagoon. Eleven sites, each about 30 m apart within the Churince water system, were chosen for small scale community sampling. At any point water samples were taken carefully as to not disturb the sediment (Column samples). Then approximately 50 μL of superficial (first millimeters TOP sample) sediment were obtained, and then another approximately 50 μL sample 2 cm below this (DEEP sample) (**Figure 1**). The samples were taken to an improvised in field laboratory, and a few hours later PBS buffer was added to the sample, and it was heat treated to select for thermo-resistant bacteria and plated out on Marine Medium (MM; Cerritos et al., 2008), and incubated at 37°C for 2 days. Different colony morphotypes were selected by size, shape, and color. Isolates were purified by subculture on the same medium to ensure that the culture was axenic; all isolates were stored at -80°C in MM with 15% (w/v) glycerol. In this work we show results for 467 thermo-resistant isolates that were obtained from eleven sampling points (**Figure 1**).

### DNA Isolation, Molecular Markers and Phylogenetic Reconstruction

DNA from isolates was purified using the QuickGene DNA tissue Kit S (Fujifilm Corporation, Minato-Ku, Japan) following the manufacturer’s instructions. We amplified the 16S rRNA gene by PCR using previously published primer sequences 27F and 1492R (Lane, 1991). This included regions V1 to V3 (∼275 bp of the 5′ end region), considered to be the most informative for the *Bacillus* spp. (Goto et al., 2000). A 700 bp segment of the *gltX* gene (glutamyl-tRNA synthetase) was amplified using the primers gltX-for (5′ CGYGGBGADGAYCAYATYT 3′) and gltX-rev (5′ CRATTTCMGCDCCRWARCT 3′) and PCR-amplified by 30 cycles of 94°C for 30 s, 47°C for 1 min, 72°C for 2 min, and then a final elongation step at 72°C for 8 min with a thermocycler Palm-Cycler (Corbett Research). The PCR products were sequenced using the Sanger method (Sanger et al., 1977) at Cinvestav-Langebio (Irapuato, México). The quality of the 16S rRNA gene sequences was assessed using Phred (Ewing et al., 1998), and sequences of at least 500 bp were used for the analysis. The *gltX* gene sequences were assembled with CAP 3 (Huang and Madan, 1999). Gene accession numbers and lists of all 467 strains can be found in Supplementary Table S1. Maximum likelihood (Guindon et al., 2010) was used to reconstruct a phylogenetic history of all thermo-resistant bacteria through the 16S rRNA gene. A second phylogenetic reconstruction was done using the MrBayes program with sequences from a subsample of 138 *Bacillus* spp. for which phenotypic traits were obtained. Sequences were aligned with Muscle Software (Edgar, 2004). 16S rRNA Maximum-Likelihood tree was reconstructed with MEGA6 Software (Tamura et al., 2013). Following Bayesian Information Criteria (BIC) the Tamura 3-parameter + Gamma model was used to estimate distances between sequences on the tree generated with 100 bootstrap replicates. The 16S rRNA Bayesian tree was reconstructed with MrBayes (Ronquist et al., 2012). We used 5,000,000 Markov chain Monte Carlo generations and the GTR + G model of evolution. Average standard deviation of split frequencies dropped below 0.01. The *gltX* sequences of these 138 *Bacillus* spp. were also obtained to increase the resolution of the particular groups. Data from the group had shown that this gene allows more resolution than *gyrA* and *rpoD*. Phylogenetic reconstruction was done for these sequences using MrBayes and the sequences were grouped with CLANS (Huelsenbeck and Ronquist, 2001; Frickey and Lupas, 2004).

### Phylogenetic Community Analysis

A total of 467 16S rRNA gene sequences were analyzed (TOP 240, DEEP 164 and COLUMN 63) using Mothur to assign to operational taxonomic units (OTUs) having 97% similarity thresholds (Schloss et al., 2009). The ribosomal database project (RDP) classifier was used for taxonomic assignment, with a minimum confidence of 0.8 to record an assignment (Cole et al., 2007). To identify ecologically distinct populations we examined the phylogenetic data for the group using AdaptML software, since it classifies a phylogeny based on genetic and ecological data and identifies populations as groups of related strains with distinct environmental distributions (Hunt et al., 2008). Tree drawings were generated with iTOL (Letunic and Bork, 2011). The net relatedness index (NRI) measures the degree of phylogenetic clustering of taxa across a phylogenetic tree in a given sample relative to the regional pool of taxa and was calculated with the Picante package (Webb, 2000; Kembel et al., 2010).

### Phenotypic Assay of a 141 *Bacillus* spp. Isolate Subsample

To evaluate some phenotypic traits of the isolates, including carbon source utilization, growth kinetics, auxotrophies, motility assays, and sporulation frequency, we used a modified LPDM medium (Muller et al., 1997) that contained: Tris (pH 8.0) [50 mM], NH_4_NO_3_ [3.3 mM], K_2_HPO_4_ [0.065 mM], MgSO_4_ [3.5 mM], sodium citrate dehydrate [6.8 mM], MnCl_2_ [1 mM], ZnCl_2_ [0.01 mM], NaCl [0.17 M], FeCl_3_ [49.9 μM], CaCl_2_ [3.60 mM], KCl [0.67 mM], glucose [100 mM], vitamin B complex [100 μL/L], biotin [0.1 mg/mL], nicotinic acid [0.1 mg/mL], peptone [0.05%] and 20 amino acids (Harwood and Cutting, 1990).

To determine preferences for carbon source utilization we used five carbon sources: xylose, raffinose, sorbitol, trehalose, and glucose, all at a concentration of 100 mM in the LPDM medium. The assays were performed using microtiter plates (96 wells) and read on a BioTek μQuant Microplate Spectrophotometer. To estimate the maximum growth rate (μmax) and saturation density, growth kinetics were monitored under two concentrations of glucose (5 and 50 mM). The plates were incubated at 30°C, with constant vibration (100 Hz) and 90% humidity. Growth was monitored every 30 min for 24 h at OD_600_ using a microtiter plate reader (Tecan Infinite^®^ M1000) assisted by a TECAN EVO robot (Tecan Infinite^®^ M1000).

To evaluate whether the different strains could grow in the absence of a given amino acid, peptone or yeast extract, cultures were first grown in liquid MM, and then passed twice to LPDM lacking amino acids, yeast extract or peptone. Strains that grew under these conditions were considered to be prototrophs. Those strains that required addition of amino acids, peptone or yeast extract were further evaluated by transferring them to LPDM that contained either all 20 amino acids, and/or peptone or yeast extract. Those strains that could grow without peptone and yeast extract were then evaluated in LPDM medium containing all but one of the 20 amino acids. Growth was monitored at 24 h (Supplementary Table S2).

Motility assays were performed according to the protocol described in Hamze et al. (2009) for swarming, and Kearns and Losick (2003) for swimming. Briefly, Petri dishes were prepared with either 0.6% (swarming) or 0.3% (swimming) MM agar and allowed to dry at room temperature. Each Petri dish was inoculated at the center and the culture was allowed to grow for 24 h at 37°C. The ability to form biofilms was determined in a microtiter plate (24 wells) in MM liquid after static incubation for 48 h at 37°C.

### Phylogenetic Signals of *Bacillus* spp. Traits

We compared the phylogenetic signal for the different phenotypic traits under evaluation. We determined the *K* value (Blomberg et al., 2003), a parameter that describes whether a pattern that arises when closely related taxa are more ecologically similar to each other than to distantly related taxa, and used this value to describe a tendency (pattern) for evolutionarily related organisms to resemble each other (*K* value is used for continuous traits, such as growth). *K* = 1 indicates a Brownian expectation, in which trait changes along each branch are random, and *K* > 1 indicates traits that are more conserved than the Brownian expectation. *K* = 0 indicates absence of a phylogenetic signal, and values between 0 and 1 indicate a significant phylogenetic signal but less conserved than under a Brownian motion model of evolution. We also carried out a Fritz and Purvis *D*-test applied to traits with discrete values. A *D* < 0 suggests a highly clustered trait, *D* ∼ 0 indicates a Brownian motion mode of evolution (BM), *D* = 0 suggests a random mode of evolution and *D* > 1 suggests phylogenetic overdispersion (Fritz and Purvis, 2010). *D* statistics can also be expressed in the same terms as the *K* statistics: -*D* + 1 = 0 value does not show a significant signal, -*D* + 1 > 0 is more conserved than expected by chance, 0 < -*D* + 1 < 1 indicates that is less conserved than expected under Brownian Motion model, -*D* + 1 = 1 is as conserved as expected under Brownian Motion model and – *D* + 1 > 1 suggest that is more conserved than expected under Brownian Motion model (Goberna and Verdú, 2016). *P*_random_ and *P*_Brownian_ convey the probability that the presence of a trait is the result of either a random or a Brownian motion mode of evolution, respectively. These tests help to identify the phylogenetic conservatism. The *K* and *D* values were calculated using the software packages Picante and Caper, respectively (Kembel et al., 2010; Fritz and Purvis, 2010), and both analyses were conducted in the R software environment (R Development Core Team, 2009). We used the consenTRAIT algorithm, that allows determining the cluster size where most organisms share a trait or, in other terms, the phylogenetic depth at which traits are conserved (Martiny et al., 2013).

The statistical analysis one-factor ANOVA was used to analyze the most numerous groups of *Bacillus* such as *B. subtilis*/*licheniformis. B. pumillus. B. cereus*, group 2, and the aquatic members. The traits subjected to this analysis were: carbon source utilization, maximum growth rate, and saturation density. The statistical analysis was carried out in the R programming environment (R Development Core Team, 2009).

## Results

### Phylogenetic Relatedness Analysis Revealed More Clustering from Thermo-Resistant Isolates in Sediment Communities Than in Water Column

We first characterized the diversity of culturable thermo-resistant bacteria from 11 sites within the Churince intermediate lagoon at CCB. We recovered 467 thermo-resistant isolates and analyzed partial 16S rRNA sequences that resulted in 77 OTUs (using Mothur with 97% similarity thresholds; **Figure 1**; Schloss et al., 2009). Among the 467 isolates, 428 (91.6%) were classified as *Bacillus* spp., 26 were classified as *Koccuria* spp., and 16 as *Staphylococcus* spp. (**Figure 1**). Sequences were assigned to a habitat category: COLUMN (water column, 63 members), TOP (surface sediment, 240 members), and DEEP (sub-surface sediment, 164 members). In order to know the phylogenetic structure among the different environments sampled, we determined if there was phylogenetic clustering among the 467 isolates using NRI, an index of community phylogenetic relatedness. We obtained positive NRI values for the sediment samples (TOP with 0.58 and DEEP with 1.83) indicative of phylogenetic clustering. For COLUMN sequences, however, the NRI values were negative (-2.28), suggesting a dispersed phylogenetic pattern of the thermo-resistant isolates from water column in comparison with the sediment sample. OTUs present in the COLUMN sample were more genetically dispersed, while those from sediment are more clustered, particularly those from the DEEP sample. We examined whether there was spatial resource partitioning among thermo-resistant isolates from COLUMN, TOP and DEEP samples. To do this, we used AdaptML which uses genetic and ecological similarity and classifies populations forming a common habitat into “projected habitats.” The populations were not classified and instead the analysis identified a single projected habitat, suggesting that there is no niche differentiation (data not shown).

### Preferences for Carbohydrate Utilization among the *Bacillus* spp. Lineages

Clustering based on 16S rRNA gene revealed that several culturable *Bacillus* spp. lineages co-occur in the Churince system. To determine the phenotypic coherence of the different taxonomic lineages, we carried out a phenotypic trait analysis in a subsample of 141 isolates from 5 sampling sites. All strains in the subsample belonged to the *Bacillus* genus and represented the dominant taxa. The chosen isolates were distributed in 13 clades (according to CLANS analysis, Frickey and Lupas, 2004), of which 6 included 80% of all members. The selected isolates were phylogenetically closer to *B. subtilis/B. licheniformis. B. pumillus. B. marisflavi. B. aquimaris. B. horikoshii*, and *B. cereus.* According to a previous classification of *Bacillus* species based on evolutionary and functional relationships, we classified the isolates into three different groups: (1) soil members (*B. subtilis/B. licheniformis*, and *B. pumillus*), (2) aquatic members (*B. marisflavi, B. aquimaris*, and *B. horikoshii*), and (3) *B. cereus*-related species (Alcaraz et al., 2010).

**FIGURE 2:**
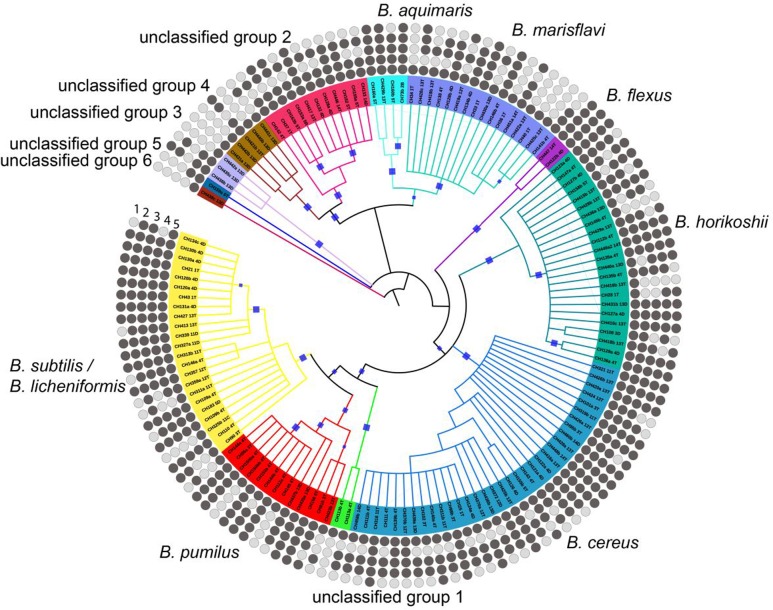
**Variability in the utilization of different carbon sources by the different *Bacillus* taxonomic groups.** Rings from the outside to the inside are designated by numbers for the carbon sources tested: (1) sorbitol, (2) raffinose, (3) trehalose, (4) xylose, (5) glucose. Dark and light circles indicate utilization and no utilization, respectively, of carbon sources. The phylogenetic reconstruction was made using 16S rRNA gene alignment and was based on the maximum likelihood method and the bootstrap values in the range from 0.8 to 1 were marked with blue squares.

The *Bacillus* spp. exhibited differences in the preference for different carbon sources. We addressed whether this was an overdispersed trait or if there was a phylogenetic signal associated to the phenotype. We chose the Purvis and Fritz’s *D* value that permits evaluating discrete traits and the significance of their genetic grouping. All isolates could grow on glucose and all clades had some members capable of using xylose, raffinose, sorbitol, and trehalose for growth (**Figure 2**). The soil member group exhibited the broadest ability to use different carbon sources, followed by the aquatic group, and finally the *B. cereus* group. All *Bacillus* spp. were able to consume glucose, so this is a highly conserved trait. On the other hand, the Fritz and Purvis’ *D* value for trehalose, raffinose and sorbitol show a significant phylogenetic signal (*D* = 0.77, 0.76 and 0.81, respectively) and differs significantly from the random and Brownian motion model expectations (*P*_random_ < 0.05 and *P*_Brownian_ < 0.05). Finally, xylose does not show a significant phylogenetic signal (*D* = 0.85), since it does not differ significantly from the random model expectations.

Since the Churince water system is an oligotrophic environment, we also evaluated the ability of *Bacillus* spp. to respond to changes in nutrient availability as well as the saturation density (maximum growth density) the community members could reach. Isolates from mainly the *B. subtilis/B. licheniformis* group responded to increased glucose concentrations, but a significant growth increase was also observed in strains that were scattered throughout the phylogeny (**Supplementary Figures S1A,B**). All groups exhibited higher saturation density at higher glucose concentrations, but there were no statistically significant differences in saturation density or maximal growth rate in either of the two glucose concentrations across the taxonomic groups (ANOVA analysis did not give a significant value for Vmax or saturation density at 5 and 50 mM glucose; **Supplementary Figure S1A**). For this data, the Blomberg *K* value from Vmax was 1.27 at 5 mM glucose, and 3.75 at 50 mM glucose, which indicates that the observed value is more conserved than that expected in a Brownian model. The Blomberg *K* values for saturation density were 0.83 and 0.56 at 5 mM and 50 mM glucose, respectively, suggesting that the traits are conserved (all *P*-values observed were significant Vs. random variance of 0.001; Supplementary Table S3B). These data suggest that Vmax growth and saturation density values are conserved traits for the different taxonomic groups.

### Most *Bacillus* spp. Isolates Are Auxotrophs, Suggesting a Strong Dependence on the Community

**FIGURE 3:**
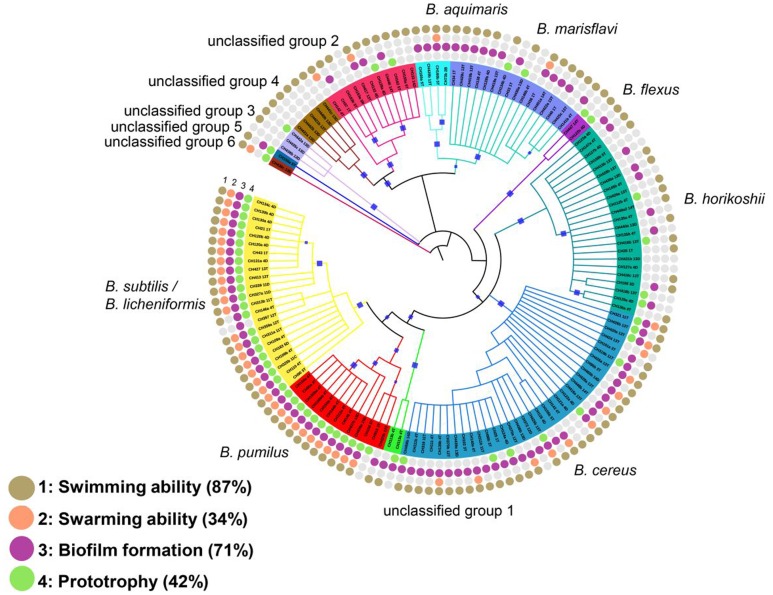
**Traits of the isolates in the different taxonomic groups of *Bacillus* spp.** Rings from the outside to the inside indicate the traits tested: (1) Swimming, (2) Swarming, (3) Biofilm and (4) Prototrophy. Filled circles indicate positive results for the described trait. Open circles indicate negative results for the evaluated trait. Bootstrap values in the range from 0.8 to 1 were marked with blue squares.

Co-occurrence of bacteria in a community could allow metabolic sharing or simply provide ample opportunity for scavenging and, either way, there would be no need for all members to be self-sufficient. We, therefore, evaluated metabolic self-sufficiency by analyzing auxotrophy for the different amino acids among members of the *Bacillus* spp. taxa from the sediment communities. Close to 60% of the evaluated *Bacillus* spp. isolates had an auxotrophy. Some members exhibited a strong dependency on peptone or yeast extract for growth even in the presence of all 20 amino acids, and this too was scored as an auxotrophic trait (44 strains required these supplements). These strains may obtain their amino acids in the form of peptides. For example, growth of aquatic *Bacillus* spp. (*B. horikoshii. B. aquimaris*, and *B. marisflavi*) exhibits extensive dependence on added amino acids or peptone (**Figure 3**). The same is true for *B. cereus*, for which prototrophy was rarely observed. The analysis of phylogenetic signal for this trait was consistent when measured with K and D statistics. It is compatible with a Brownian model of evolution using Bloomberg’s analysis (K of 1.28 with a PIC_random_ and a *P*_Brownian_ of 0.0009) implying a non-random phylogenetic signal. The D statistic was 0.27 with a *P*_random_ = 0 and *P*_Brownian_ = 0.12, a pattern that differs significantly from the random model, but the Brownian Motion model of evolution can not be rejected (Orme, 2013). Analysis with calculated a depth for 16S of τ_D_ 0.0095 16S rRNA distance, deeper than those computed for individual sugars (τ_D_ range from 0.001 to 0.003 16S rRNA distance), suggesting deeper lineage conservation (Supplementary Table S3C).

### Microdiversity in Social Traits of the *Bacillus* spp. in Sediment Communities and a Phylogenetic Signal Compatible With a Brownian Model of Evolution

We determined the frequency of swimming and swarming motility and the ability to form biofilm in this *Bacillus* spp. collection. Our results showed microdiversity for these traits, but more so in certain taxonomic groups. Although swimming seems to be a conserved trait among all clades, some members in the aquatic group seem to have lost this feature. For example, 9 out of 23 *B. horikoshii* strains could not swim (**Figure 3**). Notably, swarming was exhibited by most members of the *B. subtilis*/*licheniformis* and *B. pumilus* clade, but 75% of *B. cereus* members scored negatively for this feature (22 of a total of 34). Swarming was absent in all members of the *B. horikoshii* clade and was seen in only one member of the *B. marisflavi* and *B. aquimaris* clade (**Figure 3**).

**FIGURE 4:**
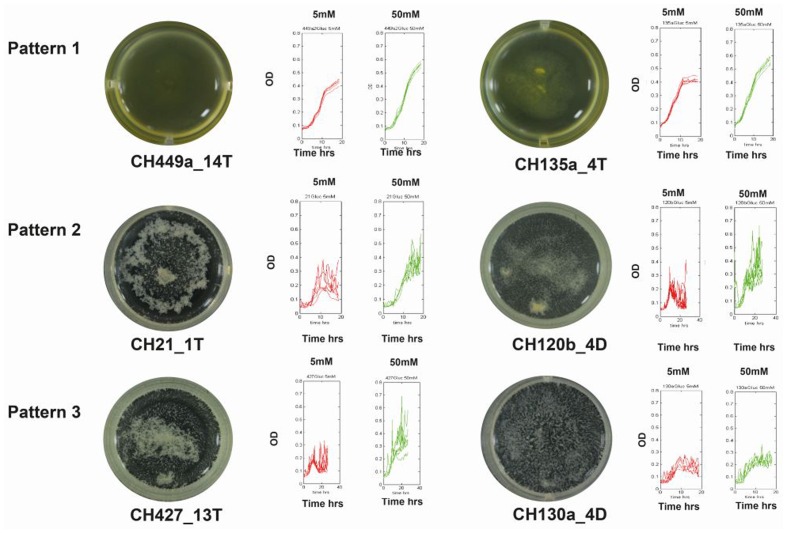
**Relationship between growth patterns and biofilm production.** Graphic representation of the three typical growth curve patterns at two glucose concentrations. The red and green curves describe growth with 5 and 50 mM glucose, respectively. Photographs at the ends of the figure depict strains at the end of the growth curve for each pattern.

For biofilm formation, heterogeneity was most noticeable among the aquatic *Bacillus* spp., but this trait was almost always present in members of the *B. subtilis/licheniformis-pumilus* or *B. cereus* groups (**Figures 3** and **4**). Moreover, almost all soil group members could form biofilms (only 1 of 36 members of the *B. subtilis*/*licheniformis. B. pumilus* group did not, and 3 out of 34 of the *B. cereus* members did not display this feature) but less than half of the *B. horikoshii* clade formed biofilms.

Growth kinetics analyses for the different isolates revealed three different but consistent patterns for the assayed *Bacillus* strains: Pattern 1, a characteristic smooth growth curve was present throughout the growth period (47% of the isolates); Pattern 2, a smooth logarithmic growth phase was followed by aggregation at the end of growth period (resulting in fluctuating readings during the stationary phase in 50% of the isolates); and Pattern 3, readings fluctuated throughout growth (this pattern occurred for 3% of the evaluated strains) (**Figure 4**). These patterns indicate that there were differences in the growth strategies of the different strains, wherein some could display a planktonic behavior (Pattern 1) with no aggregation at the end of growth (although our evaluation for biofilm formation showed that many strains had this ability) and some had aggregation that was triggered immediately at the end of the exponential growth phase (Pattern 2).

Analysis of phylogenetic signal showed that the distribution of biofilm-forming ability across the taxonomic groups of *Bacillus* spp. from these sediment communities exhibited only a moderate level of phylogenetic clustering (*D* = 0.51), and differs from a random and Brownian model (*P*_Brownian_ 0.02 and *P*_Random_ 0). *K* is 0.8 and is significantly different from random (Supplementary Tables S3A,B). We conclude that for this trait there is a departure from a random model and a Brownian model of evolution. Regarding swimming, the *D* value obtained suggested that this phylogenetic exhibited a random mode of evolution (*D* = 0.90; *P*_Random_ = 0.19, *P*_Brownian_ = 0). A Blomberg analysis, in contrast, resulted in a *K* = 0.78 with a *P* of 0.0009 for a non-random mode of evolution. For swarming and prototrophy the *D* values were 0.21 and 0.28, respectively. The statistic indicates that both swarming and prototrophy traits differed from a random pattern of evolution and resembled a Brownian motion (both have a *P*_Random_ = 0; swarming *P*_Brownian_ = 0.181, prototrophy *P*_Brownian_ = 0.106).

We also computed the phylogenetic depth at which the observed traits were conserved using consenTRAIT (Martiny et al., 2013). It calculates the mean depth of clades containing above 90% of members sharing a trait. Cluster size is therefore an important data to take into account for this analysis. For all traits evaluated the 16S rRNA distance was τ_D_ = 0.00073 to 0.0095 16S rRNA distance. As a reference, the deepest value, τ_D_ 0.07416S rRNA distance, would be that for glucose (used by all the strains in the study). Lower distances (τ_D_) were obtained for substrate utilization than for social traits (except for swarming and xylose). The largest size clusters computed were for prototrophy, swimming, and biofilm formation (size 38), compared to swarming (size 23), but the mean cluster size was higher for the social traits (biofilm, swimming, prototrophy). Social traits also had more depth signal (for biofilm, swimming, prototrophy τ_D_ = 0.005, 0.0056, 0.0095, 16S rRNA distance respectively) compared to the genetic distance for traits involved in substrate utilization (i.e., trehalose τ_D_ = 0.0011 and raffinose τ_D_ = 0.0015 16S rRNA distance) (Supplementary Table S3C).

Overall, the evolutionary models that describe the distribution of social traits in the phylogeny (except for swimming) suggest a Brownian model of evolution and a deeper phylogenetic signal than that observed for substrate utilization.

### Colony Morphology and Pigmentation Are Highly Conserved, Clade-Specific Traits

Colony morphology, size, and pigmentation were observed to be very conserved and are distinctive features of some clades. The colony appearance in our sample is a good proxy for the phylogenetic membership of a strain. Although colony color may exhibit differences in intensity and can change with growth stage and medium, color is generally consistent for members of the same clade. Colony morphology and color probably involve a large number of genes in cellular processes. These genes are differentially and spatially expressed to form a highly organized and structured community. The fact that morphology conservation occurs suggests that it is not neutral but instead arises from pressure to maintain a given developmental pattern that may be relevant for survival. However, this would have to be further studied.

**FIGURE 5:**
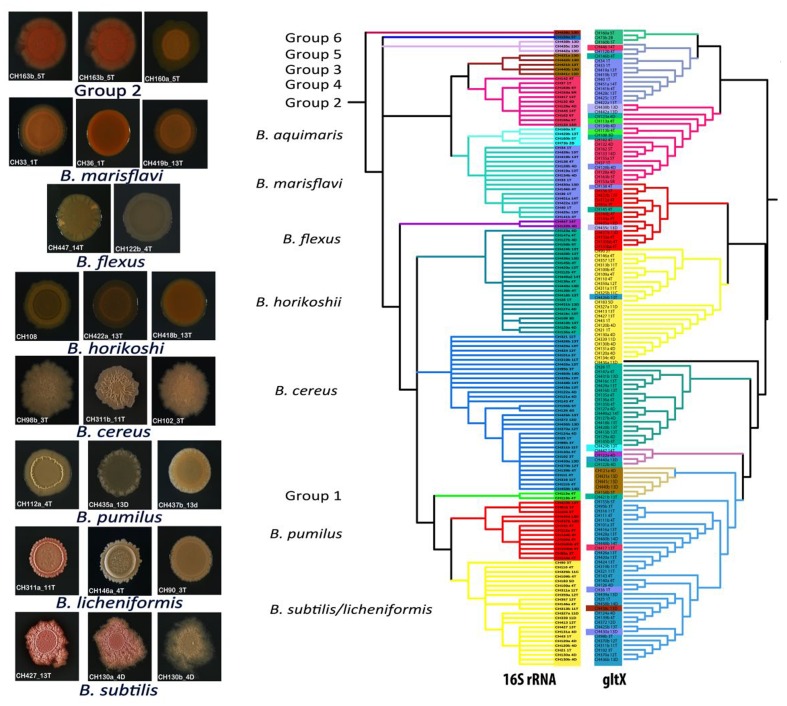
**Distinct colony morphology of members of different *Bacillus* taxonomic groups and comparison between 16S rRNA gene and *gltX* gene phylogenies.** The colored rectangles indicate strains that have moved within the phylogenetic group in the tree of 16S rRNA genes with respect to the *gltX* gene. On the left side of the figure, photographs of colonies of strains belonging to the same taxonomic clade.

Finally, the 16S rRNA gene phylogeny was coherent with respect to the phenotypes of the clade members. We added one more gene, *gltX*, to increase phylogeny resolution. As expected, this gene maintained the deeply rooted clades in the same order as the 16S, although some of the tips of the tree changed position within their own basal clade. The so-called soil *Bacillus* (*B. subtilis*/*licheniformis* and *B. pumilus)* as well as *B. cereus* members remained as a group. Meanwhile several of the aquatic groups changed position at the tips of the tree (**Figure 5**) suggesting that gene transfer is more common in the aquatic clades than in soil clades’ members.

## Discussion

Herein, we addressed individual phenotypic trait heterogeneity in members of *Bacillus* spp. co-occurring in sediment communities in an extremely oligotrophic environment and compared the phylogenetic signal associated with substrate utilization and social phenotypic traits.

The *Bacillus* spp. collection in this study provided a strong model to explore trait variability because, as we have shown previously, its assemblage in the sediment community depends more on ecological interactions than on abiotic factors (Pérez-Gutiérrez et al., 2013). Our data showed ecologically phylogenetic clustering of the sediment samples, compared to those of the water column. Sediment and water may be acting as habitat filters (Webb et al., 2002) explaining the clustered structure of the soil taxons. A similar observation was made by Rebollar et al. (2012) in a study of *Exiguobacterium* genus sampled from different ponds at CCB that found ecologically differentiated clusters associated with either sediment or water environments. These results are also consistent with those from Burke et al. (2011) showing that bacterial communities associated with *Ulva australis*, an alga found in tidal pools, differed from those found in sea water.

Unpublished data from our group has shown that upon plating, two orders of magnitude more colonies belonging to *Bacillus* spp. are consistently recovered from sediment than from water column. The fact that sediment seems to be the preferred habitat for the *Bacillus* from the lagoon under study could imply a preference for spatially structured communities with a predictable neighborhood. This is consistent with the idea that bacteria in nature predominate as adherent populations (Costerton et al., 1995.). This arrangement would not be unexpected since biofilms are thought to represent the dominant state of bacteria in nature. Biofilms are extremely complex structures and, in the particular case of *B. subtilis*, up to five cell types have been shown to contribute to the spatial organization and function of biofilms: motility, sporulation, and production of diverse matrix molecules, surfactants and proteases (Vlamakis et al., 2013).

Despite the apparent preference for sediment of the different *Bacillus* taxa evaluated and that all taxa have members with lineage-derived social traits, we observed heterogeneity in the social trait distribution at group (species) level. What does this microdiversity mean for the ecological strategies of the different *Bacillus* taxonomic groups in the community? Our evaluation of individual isolates, representing seven species within the *Bacillus* genus, revealed that for the different clades there were differences in the number of isolates scoring negatively for social traits, biofilm, swimming, and swarming, and suggests that within the same genus, different species co-occurring in the same environment (sediment communities in an oligotrophic water system) have different ecological strategies. Members of the *B. cereus* group exhibited scattered swarming capabilities and dependency on the community to obtain amino acids; in this clade, biofilm formation is a trait that is conserved in almost every member, thus stressing the importance of this strategy for the group. On the other hand, well-known members of the *B. subtilis*/*pumilus* and *licheniformis* group rarely score negatively for a social trait. This robustness could allow members of this group to move between communities and in different environments. In contrast, some members of the more endemic and local *B. horikoshii* clade do not exhibit swimming, biofilm formation, and prototrophy, and swarming is completely absent. Thus, this group would have to rely on members of the community for nutrition and perhaps for movement as well.

As reported before, most functional traits are phylogenetically conserved in bacteria (Martiny et al., 2013; Goberna and Verdú, 2016). For our data, the Fritz and Purvis distance analysis, when applied to social traits (biofilm and swarming) calculated a *D* probability closer to a Brownian phylogenetic evolutionary model. Similar to what Martiny et al. (2013) reported, all *D* values for substrate utilization were between 0 and 1, exhibiting dispersion and falling between a random and a Brownian model of evolution. A Bloomberg analysis to detect phylogenetic signal resulted in *K* value > 1 only for some traits related to substrate utilization (sorbitol, and trehalose) suggesting that those traits were clustered, that is, with closely related species being more similar than expected by chance. However, for other substrates (xylose and raffinose) and for the social traits (swimming, swarming and biofilm), *K* was below 1, though still more similar to a Brownian model than to a random model. With both measures phylogenetic signal was significantly different from random for the two most relevant social traits, swarming and biofilm, suggesting that these exhibited microdiversity but were not phylogenetically dispersed. The data obtained with consenTRAIT resulted in lower distances (τ_D_) for substrate utilization than for social traits (except for swarming and xylose), and the clusters’ mean was higher for social traits, suggesting that despite exhibiting microdiversity there is a stronger phylogenetic signal for these social traits than for substrate utilization. From what it is known about the Biology of the *Bacillus* genus, the evaluated traits can be traced back in the lineage to phylum level (Firmicutes) but are also present in several phyla (Kearns, 2010). It is intriguing why these traits exhibit a high intraspecific variation.

Differentiation in the ability to use various carbon substrates was expected and explains why numerous bacteria can co-exist in a community. Substrate utilization depends on only on few genes and is thus easily transferred (Martiny et al., 2013). Similarly, we have consistently observed microdiversity in traits for phosphorus scavenging in a large sample of *Bacillus* spp. from the CCB (Tapia-Torres et al., 2016). But, why is the phylogenetic signal of complex traits, that requires numerous genes, similar to that observed for simple traits (few genes)? An elementary explanation could be that expression of these complex traits finally depends on simple traits: sugars, such as exopolysacharides, in the case of biofilm, surfactin, for swarming, etc. If simple genes are behind the control of social (complex) traits, the signal will resemble that observed for the distribution of genes encoding enzymes involved in substrate utilization.

There are at least two explanations for what seems to be the intra-specific loss of traits: (i) real loss due to complete dispensability of the trait, and (ii) compensated loss due to the availability of public goods. For the first explanation, some traits would be lost by genetic drift and mutation because natural selection does not maintain them. For example, under the water-abundant conditions of sediment, movement by swimming might allow sufficient mobility for most cells and thus swarming might be dispensable. However, this situation can also be explained by compensated trait loss, as the addition of surfactants has been reported to complement bacteria that are defective in swarming (Kearns and Losick, 2003). Compensated trait loss (Visser et al., 2010) is promoted by extensive sharing in the community, as that occurring extensively in biofilms. Ellers et al. (2012) provide a useful framework to distinguish between the different terms that describe the evolutionary dynamics of interacting organisms. The “black queen hypothesis” (Morris et al., 2012) suggests that gene loss is adaptive in oligotrophic environments since essential functions are shared. Although we observed loss of different phenotypes, fitness analysis with non-isogenic strains complicates testing of this hypothesis. Also, we did not observe increased growth rates that were associated with the loss of phenotypic traits, which would have suggested a trade-off. Compensated trait loss through species interactions has been observed in the loss of lipogenic capacity of the parasitic fungus *Malassezia globosa* that feeds on lipids found on the skin of the host (Xu et al., 2007). Another example is loss of arginine biosynthesis in two leaf-cutter ant genera (Nygaard et al., 2011; Suen et al., 2011). The extensive auxotrophy observed is consistent with the notion that compensated trait loss occurs in communities where availability of resources permits the individual loss of traits as long as the community can maintain the given function. Some traits for the *Bacillus* spp. may not have been lost but their expression may instead depend either on specific interactions or triggered by metabolites produced by the community. All clades had members that scored positive for the evaluated trait, suggesting that members that scored negatively for the phenotype have the potential to manifest the function under different conditions or upon interaction with members of the community. There is evidence of the extensive signaling that controls biofilm formation (Vlamakis et al., 2013) and many reviews have discussed the principles of social evolution in microorganisms (Crespi, 2001; Foster et al., 2006; West et al., 2006; Diggle et al., 2007; de Vargas et al., 2011), some specifically focusing on biofilm (Nadell et al., 2008; Mitri et al., 2011), which has been argued to constitute an example of division of labor (van Gestel et al., 2015).

Sequencing of the genomes of the *Bacillus* spp. in this study will allow us to explore the nature of the observed phenotypic microdiversity at the genetic level, but the challenge remains of evaluating the phenotypic traits of individual bacteria in their communities and understanding how intra-species heterogeneity evolves.

## Conclusion

The different lineages of the *Bacillus* genus that co-exist in sediment communities of the Churince system are an excellent model for an in-depth study of intra-genus and intra-species trait heterogeneity. We found microdiversity in social traits suggestive of different ecological strategies of the taxonomic groups. The phylogenetic signal suggests that substrate utilization and social traits conform to a Brownian model of evolution that could be explained by distributed functions in structured communities.

## Author Contributions

MR-T contributed to the conception and design of the study, data acquisition and analysis, interpretation of the results, and preparation of the manuscript; AI-R and IH-G contributed to data acquisition and analysis; ZG-L and LD contributed to data acquisition and analysis, and to interpretation of the results; GO-A participated in the conception and design of the study, and to manuscript preparation; MT contributed to the design, discussion and critical revision of the manuscript, VS participated in obtaining sampling permits, discussion of the results and in a critical revision of the manuscript.

## Conflict of Interest Statement

The authors declare that the research was conducted in the absence of any commercial or financial relationships that could be construed as a potential conflict of interest.

## Supplementary Material

**FIGURE S1 | Growth and saturation density of the different *Bacillus* spp. analyzed by taxonomic group. (A)** Maximal growth rate was evaluated for each strain at two different glucose concentrations, 5 and 50 mM. (B) Saturation density was recorded for the different strains at 5 and 50 mM glucose. Mainly isolates from the *B. subtilis/B. licheniformis* group responded to increased glucose concentrations, but a significant growth increase was also observed in strains that were scattered throughout the phylogeny. Anova analysis did not give a significant value for Vmax or saturation density across taxonomic groups.
