# Supplementary figures and images for: Phenotypic Microdiversity and Phylogenetic Signal Analysis of Traits Related to Social Interaction in Bacillus spp. from Sediment Communities
Source: Front Microbiol. 2017 Jan 30;8:29. doi: 10.3389/fmicb.2017.00029 (PMC5276817; doi:10.3389/fmicb.2017.00029)

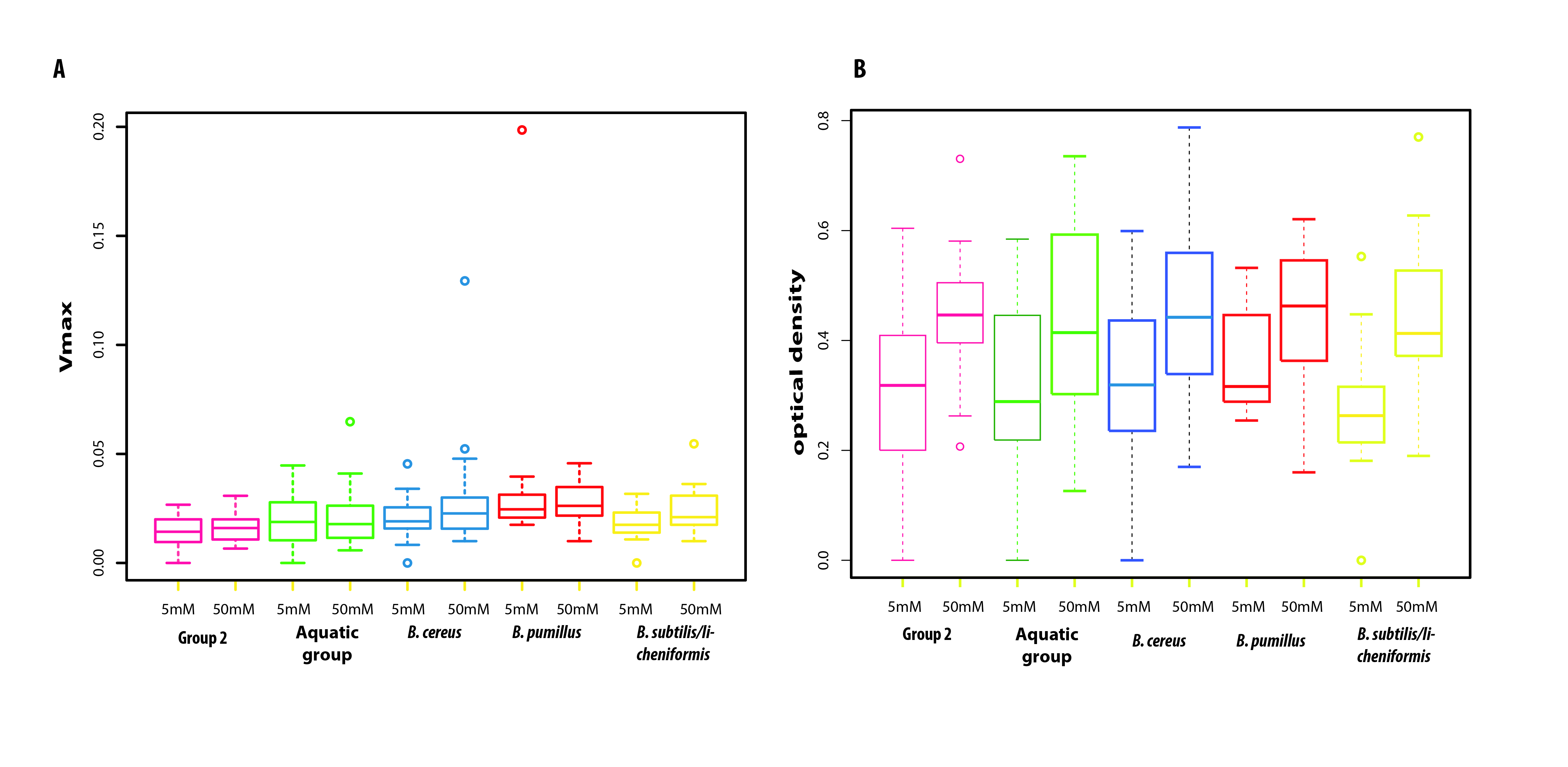

Supplement: Supplementary file 1 [file Image_1.JPEG]
