# Supplementary material for: Phenotypic Microdiversity and Phylogenetic Signal Analysis of Traits Related to Social Interaction in Bacillus spp. from Sediment Communities
Source: Front Microbiol. 2017 Jan 30;8:29. doi: 10.3389/fmicb.2017.00029 (PMC5276817; doi:10.3389/fmicb.2017.00029)
Supplement: Supplementary file 3 [file Table_3.docx]

Supplementary Table 3A. *D* statistics test on substrate utilization and social traits in the *Bacillus* spp. phylogeny

| **Trait** | **D value*** | **-D +1 (conversion to compare with K**)** | **Probability that data adjusts to a random model** | **Probability that data adjusts to a Brownian model** | **Interpretation (according to Bloomberg 2003 and Martiny et al., 2013)** |
| --- | --- | --- | --- | --- | --- |
| Prototrophy | D= 0.28 | 0.72 | P_random_= 0 | P_Brownian_= 0.12 | Clumped distribution, differs significantly from the random**, consistent with a Brownian motion** |
| Biofilm | D= 0.51 | 0.49 | P_random_= 0 | P_Brownian_= 0.016 | Clumped distribution, but differs significantly from the random and Brownian motion expectations |
| Swarming | D= 0.20 | 1.208 | P_random_= 0 | P_Brownian_= 0.192 | Clumped distribution, differs significantly from the random and is **consistent with a Brownian motion** |
| Swimming | D= 0.90 | 0.1 | P_random_= 0.19 | P_Brownian_= 0 | Clumped distribution, consistent with random model |
| Xylose | D= 0.85 | 0.15 | P_random_= 0.10 | P_Brownian_= 0 | Without significant phylogenetic signal, since it does not differ from the random expectation |
| Raffinose | D= 0.77 | 0.23 | P_random_= 0.03 | P_Brownian_= 0 | Significant phylogenetic signal but differs significantly from the random and Brownian motion expectations |
| Trehalose | D= 0.76 | 0.24 | P_random_= 0.03 | P_Brownian_= 0 | Significant phylogenetic signal but differs significantly from the random are less conserved than expected under a BM of evolution |
| Sorbitol | D= 0.81 | 0.19 | P_random_= 0.04 | P_Brown_= 0 | Significant phylogenetic signal but differs significantly from the random are less conserved than expected under a BM of evolution |

* Fritz and Purvis, 2010

D<0 suggests a highly clustered trait, D~0 indicates a Brownian motion mode of evolution (BM), D=0 suggests a random mode of evolution and D>1 suggests phylogenetic overdispersion. 0 > D >1 suggests a model between random and Brownian.

** Conversion to aid comparison to K value: -D+1 = 0 value does not show a significant signal, - D+1>0 is more conserved than expected by chance, 0<- D+1<1 indicates that is less conserved than expected under Brownian Motion model, - D+1 = 1 is as conserved as expected under Brownian Motion model and - D+1>1 suggests that it is more conserved than expected under Brownian Motion model (Goberna and Verdú, 2016).

**Supplementary Table 3B.** K value analysis of substrate utilization and social traits in the *Bacillus* spp. phylogeny

| **Trait** | **K value*** | **PIC.variance.**  **obs** | **PIC.variance.**  **rnd.mean** | **PIC.variance.P** | **PIC.variance.Z** | **Interpretation** |
| --- | --- | --- | --- | --- | --- | --- |
| 5mM_Sat | 0.834 | 0.010 | 0.103 | 0.001 | -3.464 | Exhibits non-random phylogenetic signal, is less conserved than expected under Brownian motion |
| 50mM_Sat | 0.556 | 0.010 | 0.068 | 0.001 | -3.237 | Exhibits non-random phylogenetic signal, is less conserved than expected under Brownian motion |
| 5mM_Vmax | 1.273 | 0.008 | 0.132 | 0.001 | -3.631 | Exhibits non-random phylogenetic signal, **strong phylogenetic signal** and conservatism of traits |
| 50mM_Vmax | 3.755 | 0.010 | 0.327 | 0.001 | -4.058 | Exhibits non-random phylogenetic signal, **strong phylogenetic signal** and conservatism of traits |
| Prototrophy | 1.279 | 0.010 | 0.149 | 0.001 | -3.858 | Exhibits non-random phylogenetic signal, **strong phylogenetic signal** and conservatism of traits |
| Biofilm | 0.800 | 0.011 | 0.079 | 0.001 | -3.6788 | Exhibits non-random phylogenetic signal, is less conserved than expected under Brownian motion |
| Swarming | 0.515 | 0.010 | 0.063 | 0.001 | -3.2898 | Exhibits non-random phylogenetic signal, is less conserved than expected under Brownian Model |
| Swimming | 0.782 | 0.010 | 0.0975 | 0.001 | -3.331 | Exhibits **no**n-random phylogenetic signal, is less conserved than expected under Brownian motion |
| Xylose | 0.771 | 0.010 | 0.0998 | 0.001 | -3.013 | Exhibits non-random phylogenetic signal, is less conserved than expected under Brownian motion |
| Raffinose | 0.485 | 0.011 | 0.0698 | 0.001 | -3.134 | Exhibits non-random phylogenetic signal, is less conserved than expected under Brownian motion |
| Trehalosa | 1.904 | 0.010 | 0.1946 | 0.001 | -4.207 | Exhibits non-random phylogenetic signal, strong  phylogenetic signal and conservatism of traits |
| Sorbitol | 1.180 | 0.010 | 0.1413 | 0.001 | -4.205 | Exhibits non-random phylogenetic signal, strong  phylogenetic signal and conservatism of traits |

*(Blomberg *et al*., 2003)

K = 1 indicates a Brownian expectation, in which trait changes along each branch are random, and K > 1 indicates traits that are more conserved than the Brownian expectation. K = 0 indicates absence of a phylogenetic signal, and values between 0 and 1 indicate a significant phylogenetic signal but less conserved than under a Brownian Motion.

Supplementary Table 3C. ConcenTRAIT analysis of substrate utilization and social traits

| **Trait** | **Mean trait depth, 16S rRNA distance (T_D_)^a^** | **Corresponding 16S identity^b^** | **Observed Clusters’ sizes^c^** | **Mean cluster size^d^** | **Singletons (% from 138 total)^e^** | **Positive phenotypes % (from 141 members)^f^** |
| --- | --- | --- | --- | --- | --- | --- |
| Peptone | 0.00073 | 99.8 % | 5, 2 | 1.11 | 27 (19.56) | 41.84 % |
| Sorbitol | 0.00098 | 99.8 % | 2,2,2 | 1.04 | 66 (47.8) | 51.77 % |
| Trehalose | 0.0011 | 99.8 % | 19,12, 4,3,2,2,2 | 1.57 | 57 (41.3) | 71.63 % |
| Raffinose | 0.0015 | 99.7 % | 23,9,11,4,2,2 | 1.96 | 51 (36.9) | 79.43 % |
| Swarming | 0.0026 | 99.5 % | 23,19 | 2.55 | 18 (13.04) | 34.75 % |
| Xylose | 0.0029 | 99.4 % | 11, 12, 4, 3, 2, 2, 2 | 1.45 | 58 (42.02) | 67.38 % |
| Biofilm | 0.0050 | 99 % | 38, 19, 4 | 2.31 | 38 (27.5) | 71.63 % |
| Swimming | 0.0056 | 98.8 % | 38, 19, 16, 6, 4, 2 | 2.75 | 28 (61.6) | 86.52 % |
| Prototrophy | 0.0095 | 98 % | 38, 2 | 2.72 | 20 (5.07) | 41.84 % |
| Glucose | 0.074 | 85.2 % | 138 | 138 | 0 | 99.29 % |

^a^ The data is ordered on the base of the τ_D_ 16S rRNA distance, in ascending order. The data associated with use of different carbon sources is shown in blue. Those related to social traits are in green. The datum for glucose, as a reference for the maximum depth when all 138 sequences are computed (cluster of 138, since all strains use glucose). Larger means deeper in the phylogeny.

^b^ 16S rRNA can be expressed as percent by the following conversion 1- (2 X **T_D_** ) (Martiny et al., 2013).

^c^ Cluster size describes the size of the clusters that share a given trait.

^d^ Mean cluster size was calculated including the singletons, not only the clades with 2 or more sequences.

^e^ Number of times that a trait occurred for a single sequence within a clade.

^f^ Percent of strains scoring positive for the different phenotypic traits experimentally evaluated, from a total of 141 strains.
